# Adaptation of Threat Responses Within the Negative Valence Framework

**DOI:** 10.3389/fnsys.2022.886771

**Published:** 2022-05-26

**Authors:** Nancy J. Smith, Sara Y. Markowitz, Ann N. Hoffman, Michael S. Fanselow

**Affiliations:** ^1^Department of Psychology, University of California, Los Angeles, Los Angeles, CA, United States; ^2^Staglin Center for Brain and Behavioral Health, Los Angeles, CA, United States; ^3^Department of Neurosurgery, University of California, Los Angeles, Los Angeles, CA, United States; ^4^Department of Psychiatry and Biobehavioral Sciences, University of California, Los Angeles, Los Angeles, CA, United States

**Keywords:** fear, anxiety disorders, defensive behavior, threat imminence, COVID-19, RDoC

## Abstract

External threats are a major source of our experience of negatively valanced emotion. As a threat becomes closer and more real, our specific behavior patterns and our experiences of negative affect change in response to the perceived imminence of threat. Recognizing this, the National Institute of Mental Health’s Research Domain Criteria (RDoC) Negative Valence system is largely based around different levels of threat imminence. This perspective describes the correspondence between the RDoC Negative Valence System and a particular neurobiological/neuroecological model of reactions to threat, the Predatory Imminence Continuum (PIC) Theory. Using the COVID-19 pandemic as an illustration, we describe both adaptive and maladaptive behavior patterns from this perspective to illustrate how behavior in response to a crisis may get shaped. We end with suggestions on how further consideration of the PIC suggests potential modifications of the negative valence systems RDoC.

## Introduction

**FIGURE 1:**
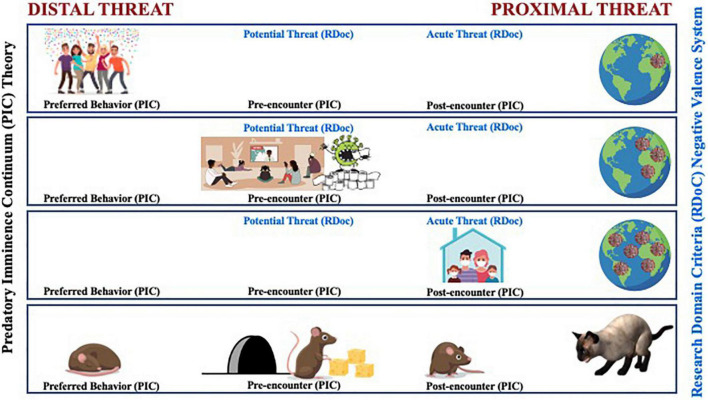
A comparative illustration between the predatory imminence continuum (PIC) theory and the RDoC’s negative valence system’s representation of changes in defensive action patterns based upon the perceived psychological proximity to the COVID-19 virus. Early in the pandemic, the COVID-19 threat was considered distal and not something to be that concerned about. Social distancing was not part of the typical behavior; instead, people engaged in more preferred social interactions (top row). As coronavirus cases surged and regular reports of hospital admittance and death tolls rose, social interaction was replaced with stockpiling supplies in preparation for the potential and pre-encounter of the coronavirus (middle row). As the threat became more proximal, stay-at-home orders were put into effect as a way to help evade contact and increase survival (bottom row). Some aspects of the evolution of the imminence of COVID-19 from distal to proximal threat also follow patterned stages in the RDoC Negative Valence System that ranged from potential threat, where increased vigilance emerges under conditions of uncertain safety, to acute threat activated when a specific danger is detected.

Beginning in late 2019, a common threat unfolded before humanity. Our reactions to COVID-19 provide an all too real illustration of how our actions are organized by the imminence of threat as it moves closer, as illustrated in Figure 1. For those in California, the sequence looked something like this:

December 2019-early January 2020: Large gatherings of friends and families pack office parties and homes as the prototypical holiday season comes and goes. We engage in our typical pattern of ***preferred activity.*** Still, the world watches with one eye the unfolding of a mysterious coronavirus-related pneumonia spread in a populated city in China. For those in distal regions, thoughts of good will are sent out to those affected, along with internal thoughts of relief, “good thing it’s not here, and it’s not me.”

Late January-early March 2020: The presence of the new disease extends beyond the borders of the point of origin with no signs of containment. Coronavirus cases rise rapidly, and regular reports of death tolls in the most affected areas catch the media’s full attention worldwide. Cases spread in distributed pockets around the world. Hospitals in the highest affected areas are overwhelmed. While as individuals, we have not directly encountered the virus, even ***pre-encounter*,** our behaviors change in preparation for the potential arrival of the virus in our community. Households send out one brave member at a time to stock up on survival supplies like toilet paper and anti-bacterial products and clear store shelves of canned and frozen foods. The stockpiling of supplies goes beyond rational and necessity.

March 2020 and Beyond: The World Health Organization declares COVID-19 a pandemic. Our communities have now directly encountered the virus. The ***post-encounter*** world shuts down in a rapid crash of dominoes. Stay at home orders affect everyone as the presence of the threat in nearly every community is confirmed. Simple daily tasks like grocery shopping or even leaving the house put one at risk of contracting COVID-19 and potentially dying.

The evolution of the imminence of COVID-19 from distal to proximal threat followed patterned stages that ranged from low to high risk to even interaction with the threat itself. As the spatial, temporal, and psychological distance from the virus narrowed, defensive response strategies shifted in an effort to evade contact and increase survival. The COVID-19 global pandemic tells a directly relatable and unavoidable story of the predatory imminence continuum.

It is important to acknowledge that the COVID-19 global pandemic remains an acute source of loss and trauma for too many. The current perspective relates the predatory imminence continuum as one way to conceptualize the complex pattern of human behavior in response to the pandemic. We acknowledge that many of the subjective emotions experienced in response to and throughout the course of the pandemic and beyond may be explained by other psychological theories, as well.

## The Predatory Imminence Continuum Theory

Animals translate environmental threat into specific patterns of behavioral action that have a phylogenetic history of protecting the species (Bolles, 1970). Supportive physiological changes accompany these species-specific defense reactions (SSDRs). Under threat, behavior becomes limited to these SSDRs, and the animal must strategically select the most appropriate SSDR from its repertoire of defensive behaviors. An influential model of SSDR selection applied to both humans and rodents is the Predatory (or Threat) Imminence Continuum (PIC) theory (Fanselow and Lester, 1988; Bouton et al., 2001; Mobbs et al., 2007, 2015). PIC states that qualitatively distinct defensive behaviors are matched to the psychological distance from physical contact with a life-threatening situation. For example, rodents freeze when they detect a predator but show vigorous bursts of activity to contact with the predator (Fanselow and Lester, 1988). Each defense mode across the PIC has unique antecedent conditions, and engages a distinct set of consequent behaviors. The three defense modes, pre-encounter, post-encounter, and circa-strike, map well onto states of anxiety, fear, and panic in mammals’ behavior (Mobbs et al., 2009; Perusini and Fanselow, 2015). Each mode is served by different, but interacting, neural circuits (Fanselow, 1994).

This organization of defensive behavior is highly conserved across many animal species, including humans (Roelofs et al., 2010; Hagenaars et al., 2014; Roelofs, 2017). The application of this framework to human defensive behaviors underscores the use of this model in understanding conditions that select for states of heightened anxiety and even the development of post-traumatic stress disorder (PTSD), phobias, and panic disorder. For example, a response to a minor inconvenience may be amplified and even trigger a panic attack (circa-strike) when in a state of already high anxiety (pre-encounter defense) compared to our ability to brush it off when presented in a neutral or relaxed state (Bouton et al., 2001).

While the PIC theory was developed from the perspective of prey-predator interactions, the organization of the PIC theory can be generally applied to environmental threats. A rat responding to a cat or a human responding to the threat of COVID-19 both have their behavior organized with respect to the spatial, temporal, and probabilistic relationship with the threat. One possibility is that the highly significant threat of being eaten by a predator was the selective force behind the evolution of the PIC. Evolution often borrows existing adaptations in a process referred to as *exaptation* (Gould and Vrba, 1982). With this defensive brain-behavior mechanism in place, other threats acquired the ability to tap into this system (exaptation) over the course of phylogeny and, perhaps even, ontogenetic experience with other types of threat. This idea of novel threats tapping into the already evolved antipredator defensive system is common and is likely why rats’ reactions to innate threats like predators and novel learned threats like stimuli predicting electric shock are highly overlapping (Bolles, 1970; Lester and Fanselow, 1985).

## Pre-Encounter Defense: Risky Foraging, Panic Shopping, and Hoarding

Despite the repeated assurance that food and supplies were not scarce or at risk for running out in our communities, panic shopping was a real phenomenon that plagued grocery and supply stores all over the world (Hao et al., 2020; Tyagi et al., 2020; Valaskova et al., 2021; Prentice et al., 2022). Pre-encounter defense consists of patterns of behavior that include increased vigilance and risk assessment. Additionally, meal pattern reorganization and cautiously leaving the nest area are components of pre-encounter defense. In early experiments simulating a naturalistic environment where foraging occurred under a low probability of threat, rodents lived for extended periods in a “closed economy” ecosystem that included a safe nest, as well as a foraging area that required lever press behavior in order to procure food (Fanselow et al., 1988; Helmstetter and Fanselow, 1993). Predation was modeled by the risk of delivery of footshock while foraging (Fanselow et al., 1988; Helmstetter and Fanselow, 1993). In these studies, the administration of random but rare shocks caused rats to *decrease meal frequency* but *increase meal size* such that while energy demands were met, the animals adapted their behavior to reduce the risk of exposure to shock by reducing total time foraging on the potentially dangerous grid. Interestingly, this meal pattern reorganization changed as a function of shock density (Fanselow and Lester, 1988; Fanselow, 1989; Helmstetter and Fanselow, 1993). In other words, as risk increased, the number of foraging trips decreased whereas meal size increased.

Similarly, during the pandemic, as the perceived risk of danger increased, humans adjusted foraging patterns. These shifts included adapting to optimal time windows for grocery shopping, relying on Google Maps data to determine the least busy store hours, implementing senior citizen shopping hours to protect the vulnerable, and a new surge in the reliance on grocery delivery. Based on PIC’s theoretical framework, one study directly tested this pre-encounter defense feature by asking whether the perceived threat of COVID-19 accounted for changes in purchasing behavior (Schmidt et al., 2021). Consistent with meal reorganization outcomes observed in the closed economy rodent experiments, Schmidt et al. found that the perceived threat of COVID-19 was a significant predictor for subjective changes in purchasing behavior, where high perceived threat was associated with *increased* purchasing quantities and a *reduction* in purchasing frequency (Schmidt et al., 2021). People faced the potential risk of COVID-19 by adopting shopping strategies similar to rats in the closed economy. Factors associated with the perceived threat of COVID-19 included higher levels of intolerance of uncertainty and high levels of media exposure, also related to increased purchasing quantity, contributing to the complexity of human threat perception during the pandemic.

Consistent with other infectious disease outbreaks and other crises such as natural disasters and extreme weather, COVID-19 led to hoarding or stockpiling behavior (Micalizzi et al., 2020). Stockpiling behavior is thought to stem from a response, either rationally or psychologically, to scarcity and psychological uncertainty (Micalizzi et al., 2020). In one survey study during the COVID-19 pandemic, the most hoarded item was toilet paper (Micalizzi et al., 2020). Additionally, high rates of guns and other weapons were procured. Interestingly, stockpiling behavior was more commonly observed in those that socially distanced less.

## Post-Encounter Defense: Freezing and Lockdown

While pre-encounter defense occurs when no specific threat is close, things change dramatically when a threat has been detected in the vicinity. One example is when a foraging rat detects a predator. The dominant response of the rat at this point is to freeze. This response is not simply a lack of movement as the rat will move toward and freeze in corners and next to nearby objects. That is, freezing and thigmotaxis are highly related (Grossen and Kelley, 1972; Fanselow and Lester, 1988). If the prey has not been detected yet, freezing is effective because movement is a visually salient stimulus that will attract attention (Rokszin et al., 2010). However, freezing is also effective when the prey has been detected because movement is often a releasing stimulus for an attack (Suarez and Gallup, 1981). While the formal study of freezing has been predominantly studied in rodents, there have been laboratory examples of freezing in humans (Roelofs et al., 2010).

The threat from COVID-19 dramatically increased when it was detected in our communities. The virus was no longer a distal threat when it was encountered. Rather than shopping trips to hoard items, people refused to leave their houses. A sort of post-encounter freezing at home because the threat was in the immediate vicinity.

A large body of literature on rodents indicates that the amygdala is intimately related to freezing (Kim et al., 1993; Fanselow and LeDoux, 1999; Fendt and Fanselow, 1999; Davis and Whalen, 2001; Fanselow and Gale, 2003). The amygdala becomes more active during freezing, and inhibition of the amygdala dramatically reduces freezing (Helmstetter, 1992; Zelikowsky et al., 2014; Liu et al., 2022). The onset of the COVID-19 pandemic allowed itself a rare opportunity for a real-life natural experiment. As part of a pre-registered study, one research group in Israel compared brain volumetric changes in healthy people who received an MRI before and after the COVID-19 outbreak and lockdown (Salomon et al., 2021). While none of the participants were physically infected with the virus, they found significant impacts of the initial lockdown period on the amygdala. The authors showed prominent bilateral increases in amygdala volume in subjects that were scanned prior to and after lockdown (March-May 2020) compared to those that received two separate scans prior to COVID-19. Interestingly, the amygdala changes showed a time-dependent effect where the biggest increases were related to the time since lockdown and not the time since the baseline scan (Salomon et al., 2021).

## The Relationship Between Predatory Imminence Continuum Theory and the Negative Valence Research Domain Criteria

With the Research Domain Criteria (RDoC) initiative, the National Institute of Mental Health of the United States (NIMH) set out to establish a framework that more tightly related mental disorders to neurobiology (“Definitions of the RDoC Domains and Constructs”) than the traditional framework described in the Diagnostic and Statistical Manual of Mental Disorders (DSM-5, American Psychiatric Association [APA], 2013). United States health care professionals use the DSM-5 as a tool to assess clinical symptoms, and clusters of symptoms due to the comorbidity, of many mental disorders. However, a gap exists between clinical research and neurobiological mechanisms that may contribute to psychopathology (Insel et al., 2010; Sanislow et al., 2010). A goal of the RDoC framework was to bridge the gap between clinical research and behavioral neuroscience (Sanislow et al., 2010; Nicholson and Sommer, 2018) to form a more precise, quantifiable understanding of neuropsychiatric diseases (Nicholson and Sommer, 2018; Pacheco et al., 2022). The RDoC may also provide a framework for improving translational relevance from preclinical to clinical research (Insel et al., 2010; Nicholson and Sommer, 2018). Within the Negative Valence System, three of the constructs were specifically related to threat: Potential Threat, Acute Threat, and Sustained Threat (see Table 1). The clear correspondence of the potential threat and acute threat constructs with the predatory imminence modes of the pre-encounter defense and the post-encounter defense emerged from the discussions at the NIMH that generated the negative valence RDoC:^1^ National Institute of Mental Health [NIMH] (2011).

**TABLE 1:** The organization of PIC defense modes and relevant RDoC constructs.

PIC Modes of defense ⇢	Pre-encounter	Post-encounter	Cirea-strike	
Predatory behavior made ⇢	Foraging	Search and procure	Handling and consumption	
Function of defensive mode ⇢	Reduce the likelihood of encountering a predator.	Decrease the likelihood of detection and attack	Survive direct contact with a predator.	
State ⇢	Anxiety	Fear	Panic	
Antecedent stimuli ⇢	Past experiences with predation or theats.	Detection of a predator or signal for imminent threat.	A striking predator is making or is about to make physical contact.	
Consequent behaviors ⇢	Stretched approach, alterations in meal patterns (less frequent larger meals), retreat to nest.	Freezing and thigmotaxis	Audible vocalization (scream), vigorous escape attempts. Protean movement, jumping	Stress causes a sustained distortion of the PIC.

**RDoC construct ⇢**	**Potential threat**	**Acute threat**		**Sustained threat**

	Activation of a brain system in which harm may potentially occur but is distant, ambiguous, or low/uncertain in probability.	Activation of the brain’s defensive motivational system to promote behaviors that protect the organism from perceived danger.	RDoC did not include a panic-like category.	An aversive emotional slate caused by prolonged (i. e., weeks to months) exposure to internal and/or external conditions(s), state(s), or stimuli that are adaptive to escape or avoid. The exposure may be actual or anticipated.
Consequent behaviors ⇢	Enhanced risk assessment (vigilance).	Normal fear involves a pattern of adaptive responses to conditioned or unconditioned threat stimuli (exteroceptive or interoceptive)		The changes in affect, cognition, physiology, and behavior caused by sustained threat persist in the absence of the threat and can be differentiated from those changes evoked by acute threat.
	These responses to low imminence threats are qualitatively different than the high imminence threat behaviors that characterize fear.	Fear can involve internal representations and cognitive processing and can be modulated by a variety of factors.		

*The PIC contains three modes of defense, which are reliant on the detection and proximity of a predator or threat. Pre-encounter, which maps on to the potential threat RDoC construct, occurs when in a situation wherein danger is distant or ambiguous and cautious behavior is necessary. The PIC theory states that this stage is analogous to anxiety. Post-encounter occurs when a concrete threat, such as a predator, has been detected. This elicits fear behavior such as freezing and thigmotaxis. Within the RDoC, this is analogous to acute threat. The PIC includes a circa-strike stage in which the predator or danger has made or is about to make physical contact and death is imminent. This panic-like stage elicits distinct behaviors such as audible vocalization, flight, protean movement, and jumping. In contrast, the RDoC includes no such category. Instead, its third category, sustained threat, refers to an aversive emotional state caused by prolonged stress or aversive stimuli. Although the PIC does not currently encompass sustained threat, we believe that significant stress causes a distortion of the PIC, which can persist even in the absence of a significant stressor (Hoffman et al., 2022). RDoC wording adapted from “Definitions of RDoC Domains and Constructs” (https://www.nimh.nih.gov/research/research-funded-by-nimh/rdoc/constructs/negative-valence-systems).*

Pre-encounter defense emerges when an organism leaves a situation of relative safety to one that has the potential of encountering a threat, making the antecedent causes of these two constructs synonymous (potential threat). A specific threat is not present, but behavior is altered in ways to reduce that potential. Behavioral changes such as increased vigilance emerge, but as we described above, under conditions of uncertain safety, there are more complex changes such as alterations of foraging patterns in both humans and rodents. The Pre-encounter/Potential Threat constructs well to an anxious state.

Similarly, the Acute Threat category was designed to correspond to Post-Encounter Defense. Both are activated when a specific danger is detected, and harm is far more likely than in the Pre-encounter mode. Given that the threat is actual, the state corresponds to fear. It is important to recognize that the difference between acute and potential risk conditions is often probabilistic, the stimulus that is the source of risk may be the same. For example, a rat may engage in pre-encounter defense because there is a possibility of encountering a threat, but a cat has not yet been detected. Post-encounter defense occurs because a cat has been detected. The source of the threat, a cat, is the same in both cases, but the probability of an attack is different. Another example was provided by Helmstetter and Fanselow (1993). They reported that rats that lived in a free foraging environment decreased meal frequency and increased meal size, defending body weight when the probability of shock while foraging was low. When shock probability increased further feeding was suppressed, and the rats lost weight. So, the same stimulus, shock, produced both Pre-encounter and Post-encounter behavior, dependent on the probability of occurrence. Interestingly, the shock probability that suppressed feeding is about the same as the shock probability needed to produce freezing (Fanselow, 1989).

## Divergence of Predatory Imminence Continuum Theory and the Research Domain Criteria

There are two points of divergence between the RDoC Schema and PIC Theory. Predatory imminence includes a component called Circa-strike behavior that is not represented in the RDoC. RDoC also considers a category of sustained threat that PIC Theory does not address. We examine these two in turn.

Circa-strike responses occur around the time of actual physical contact with a threat. In rodents, freezing is replaced by vigorous bursts of activity, struggling, and vocalization designed to evade the clutches of a predator. These reactions seem akin to panic. Interestingly, in perhaps another example of exaptation, suffocation leads to similar struggling. Both circa-strike behavior to contact related stimuli and panic reactions to CO_2_ are mediated by the midbrain periaqueductal gray (Fanselow, 1991; Graeff, 2004; Spiacci et al., 2018). The final stages of predation often include the predator suffocating the prey before consumption (Greene and Burghardt, 1978; Anton and Turner, 1997). In humans, this can be modeled by CO_2_ inhalation, which causes panic-like reactions, especially in those with panic disorder (Gorman et al., 2001; Wiese and Boutros, 2019). Similarly, rodents show Circa-strike-like panic behavior in response to CO_2_ (Graeff, 2004; Spiacci et al., 2018). The absence of a panic-like category in the RDoC is notable. Given the correspondence between PIC and the Negative Valence System, this would seem like a valuable addition and should be considered in future revisions of the Negative Valence Systems RDoC.

Sustained Threat is the category in the Negative Valence System that corresponds to exposure to aversive conditions lasting weeks to months with behavioral changes that persist in the absence of threat. Cleary, such sustained reactions are maladaptive. On the other hand, PIC Theory describes adaptive behavior; defending against a threat is the phylogenetically appropriate response in threatening situations. However, if defensive behavior persists beyond the time of threat, it would reduce the amount of time for the important non-aversively motivated behaviors that characterize the preferred activity pattern. Anxiety disorders are considered disorders when they compromise normal adaptive behavior. Perhaps we can conceptualize anxiety disorders as a distortion of the PIC. Recently, we reported that prior exposure to potent stress causes an increase in behaviors across the PIC (Hoffman et al., 2022). Furthermore, these changes in behavior persisted long after the stress, in other words, they were sustained. Thus, the sustained threat construct can be viewed within PIC Theory as a long-term distortion of the continuum.

## Conclusion

In this perspective, we develop the idea that much of our negatively valanced emotion, particularly anxiety, fear, and panic, are rooted in the systems that evolved to deal with the threat of predation. Antipredator defensive behavior is organized around a threat imminence continuum anchored on one end by typical activity patterns that occur in the absence of threat and serve biologically important functions other than defense, such as feeding and mating. The other end of the continuum is terminal, the prey is consumed by the predator. The specifics of defensive behavior and their underlying emotions proceed through stages of potential threat, to actual threat, to physical contact with the threat. Constructs within the NIMH Negative Valence system correspond to these different modes of defensive action. While predation may have provided the original selection pressure for this behavior system, over phylogeny other types of threat have tapped into this pre-existing organization through the process of exaptation. We use responses to the COVID-19 pandemic to illustrate this point. The PIC Theory allows adaptive defensive behaviors to match the currently threatening situation. We suggest that some experiences, and perhaps some genetic variations, may result in a distorted PIC that limits the time available for non-threat-related behavior by increasing time in defensive states. Such distortions of the PIC may be the basis of anxiety and stress-related disorders.

## Data Availability Statement

The original contributions presented in the study are included in the article, further inquiries can be directed to the corresponding author.

## Author Contributions

NJS made Figure 1. SYM developed Table 1. All authors contributed equally to the writing of this perspective.

## Conflict of Interest

MSF is Director of Research for Neurovation Labs. The remaining authors declare that the research was conducted in the absence of any commercial or financial relationships that could be construed as a potential conflict of interest.

## Publisher’s Note

All claims expressed in this article are solely those of the authors and do not necessarily represent those of their affiliated organizations, or those of the publisher, the editors and the reviewers. Any product that may be evaluated in this article, or claim that may be made by its manufacturer, is not guaranteed or endorsed by the publisher.

## Footnotes

1
https://www.nimh.nih.gov/research/research-funded-by-nimh/rdoc/negative-valence-systems-workshop-proceedings
